# Development and validation of a claims-based algorithm to identify incidents and determine the progression phases of gastric cancer cases in Japan

**DOI:** 10.1007/s00535-024-02167-y

**Published:** 2024-11-26

**Authors:** Takahiro Inoue, Nobukazu Agatsuma, Takahiro Utsumi, Yukari Tanaka, Yoshitaka Nishikawa, Takahiro Horimatsu, Takahiro Shimizu, Mitsuhiro Nikaido, Yuki Nakanishi, Nobuaki Hoshino, Yoshimitsu Takahashi, Takeo Nakayama, Hiroshi Seno

**Affiliations:** 1 Department of Gastroenterology and Hepatology, Kyoto University Graduate School of Medicine, 54 Kawaharacho, Shogoin, Sakyo-Ku, Kyoto, 606-8507 Japan; 2 Department of Gastroenterology and Hepatology, Japanese Red Cross Wakayama Medical Center, Wakayama, Japan; 3 Department of Internal Medicine, Hino Memorial Hospital, Shiga, Japan; 4 Department of Health Informatics, Kyoto University School of Public Health, Kyoto, Japan; 5 Department of Clinical Oncology, Kyoto University Hospital, Kyoto, Japan; 6 Institute for Advancement of Clinical and Translational Science (iACT), Kyoto University Hospital, Kyoto, Japan; 7 Department of Surgery, Kyoto University Graduate School of Medicine, Kyoto, Japan

**Keywords:** Gastric cancer, Health insurance claims, Algorithm development

## Abstract

**Background:**

Although health insurance claims data can address questions that clinical trials cannot answer, the uncertainty of disease names and the absence of stage information hinder their use in gastric cancer (GC) research. This study aimed to develop and validate a claims-based algorithm to identify and determine the progression phases of incident GC cases in Japan.

**Methods:**

The gold standard for validation in this retrospective observational study was medical records of patients with incident GC who underwent specific treatments, defined by the claim codes associated with GC treatment. The algorithm was developed and refined using a cohort from two large tertiary care medical centers (April–September 2017 and April–September 2019) and subsequently validated using two independent cohorts: one from different periods (October 2017–March 2019 and October 2019–March 2021) and the other from a different institution (a community hospital). The algorithm identified incident cases based on a combination of the International Classification of Diseases, 10th Revision diagnosis codes for GC (C160-169), and claim codes for specific treatments, classifying them into endoscopic, surgical, and palliative groups. Positive predictive value (PPV), sensitivity of incident case identification, and diagnostic accuracy of progression phase determination were evaluated.

**Results:**

The developed algorithm achieved PPVs of 90.0% (1119/1244) and 95.9% (94/98), sensitivities of 98.0% (1119/1142) and 98.9% (94/95) for incident case identification, with diagnostic accuracies of 94.1% (1053/1119) and 93.6% (88/94) for progression phase determination in the two validation cohorts, respectively.

**Conclusions:**

This validated claims-based algorithm could advance real-world GC research and assist in decision-making regarding GC treatment.

**Supplementary Information:**

The online version contains supplementary material available at 10.1007/s00535-024-02167-y.

## Introduction

Gastric cancer (GC) is the fifth most common cause of cancer-related deaths and fifth most commonly diagnosed malignancy worldwide. In Japan, it ranks the third most common cancer, making it a significant public health issue [1, 2]. Clinical practice guidelines for GC treatment [3–5] have provided detailed recommendations for various therapies based on multiple clinical trials, including endoscopic resection, surgery, and chemotherapy [6–12]. However, in clinical practice, patient characteristics, such as age, sex, comorbidities, and access to medical care, are more diverse than those in clinical trials. Even in patients at the same progression phase of GC, individualized treatment may be suggested after detailed disease characterization. Furthermore, some populations are less likely to tolerate guideline-concordant care [13, 14]. This discrepancy often complicates the decision-making regarding cancer treatment for both clinicians and patients.

Real-world data (RWD), collected from clinical practice, represent actual clinical settings, in contrast to clinical trials [15, 16]. Health insurance claims data, a type of RWD, provide information on diagnoses, medical procedures, prescribed medications, and healthcare services [17, 18]. They have been used to conduct cancer-related research on various topics, such as outcome comparisons by chemotherapy sequence [19], treatment patterns [20–24], and costs [25–27], and are expected to be used in GC research as well. In particular, the claims data of incident cases contain the whole history, from diagnosis to long-term outcomes, and may contribute to resolving clinical questions that cannot be answered by clinical trials.

However, despite their advantages and widespread use, claims data have certain limitations. Disease name codes in claims are often conveniently assigned for billing purposes and do not always correspond to the names of diseases diagnosed [28, 29]. This uncertainty can hinder the accurate identification of true cancer cohorts and produce spurious results in claims-based studies. In addition, cancer stage information is not available in claims data, which limits research requiring stratification by disease progression [30].

We previously reported an algorithm developed to identify patients with colorectal cancer and their disease stages based on health insurer-based claims data [26], which records patients’ complete medical history across multiple institutions and allows for more accurate identification of patient cohorts and tracking of the clinical course. Since our report, several studies have attempted to establish validated algorithms to identify patients with incident GC using claims data [31–33]. However, the accuracy of these algorithms remains unsatisfactory due to issues such as inappropriate listing of specific treatment codes and insufficient exclusion of prevalent cases. Moreover, to the best of our knowledge, no validation studies have been conducted on claims-based algorithms to determine the disease progression phase in patients with incident GC.

This study aimed to develop a more accurate and clinically relevant claims-based algorithm to identify and determine the progression phases of incident GC cases using multi-institutional claims data. The algorithm was originally intended for application to health insurer-based claims data.

## Methods

### Study design and population

This retrospective observational study included adult inpatients and outpatients diagnosed with GC between April 2017 and February 2023 using hospital claims, cancer registry data (April 2016 to July 2024 for claims and April 2017 to February 2023 for cancer registry data), and electronic medical records (the entire period available) from three institutions: two large tertiary care medical centers (Hospitals A and B) and one community hospital (Hospital C). Hospital A was Kyoto University Hospital, Kyoto, Japan, with 1121 beds; Hospital B was the Japanese Red Cross Wakayama Medical Center, Wakayama, Japan, with 700 beds; and Hospital C was Hino Memorial Hospital, Shiga, Japan, with 150 beds (Online Resource Fig. 1).

The study protocol was approved by the Institutional Review Boards of Kyoto University Hospital (approval no. R3472), Japanese Red Cross Wakayama Medical Center, and Hino Memorial Hospital. Since the study used existing anonymized data, the need for informed consent was waived. The study information was made available on each institutional website to provide the opportunity to opt-out. This manuscript was prepared in accordance with the Strengthening the Reporting of Observational Studies in Epidemiology (STROBE) statement [34] and the REporting of Studies Conducted using Observational Routinely Collected Health Data (RECORD) statement [35].

### The gold standard for validation

In this study, the targets to be extracted by the algorithm, namely the gold standard for validation, were data on patients with incident GC, based on electronic medical records at the three participating institutions.

#### Definition of incident cases

Incident cases were defined as cases of patients who were newly diagnosed with GC, and initiated specific treatment within the index month between April 2017 and February 2023. GC cases were also considered incident if GC occurred more than 5 years after their last radical treatment. Three board-certified gastroenterologists (T. I., N. A., and T. U.) extracted the Ministry of Health, Labor, and Welfare procedure codes associated with GC-specific procedures and the claim computer processing system codes for the anticancer agents covered by insurance for GC treatment (Online Resource Table 1). These codes, including endoscopic therapy, surgical treatment with or without primary lesion resection, radiotherapy, treatment of obstruction, and chemotherapy, were defined as GC-specific treatments in this study.

#### Definition of the progression phases

This algorithm determines the disease progression phase based on the treatment process recorded on the claims. Therefore, the targeted progression phase of incident cases was defined as the progression phase after the initial series of treatments on electronic medical charts, assessed according to the disease status 1.5 years after the index month. This timeframe was chosen because the Japanese guideline recommends adjuvant chemotherapy for up to 1 year after surgery [5], and considers a maximum of 1.5 years as sufficient to complete the initial radical treatment of GC. The same timeframe was used for the palliative cases. The disease progression phases in patients were classified into the following three groups:

- Endoscopic curative group: Resection with negative margins achieved with endoscopic treatment and no metastatic recurrence
- Surgical curative group: Resection with negative surgical margins and no metastatic recurrence
- Non-curative group: Unresectable advanced GC or metastatic recurrence after initial treatment

In addition to cancer registry information, which is an established database of patients with cancer [36], claims information was also used to broadly identify “candidate patients with incident GC.” Three board-certified gastroenterologists (T. I., N. A., and T. U.) independently reviewed the patients’ medical records to ascertain incident cases and determine their progression phases. With regard to esophagogastric junction (EGJ) cancers, gastric cardia adenocarcinoma (GCA) was included in this study. Due to the retrospective nature of this study, creating a strict classification according to the established definition [37] was difficult. Therefore, adenocarcinoma at the EGJ endoscopically exposed on the gastric side was defined as GCA.

The following patients were excluded from the assessment of the accuracy of incident case identification: (1) patients referred between institutions after receiving initial specific treatment of GC, including those who were referred to our institution from another institution or vice versa; (2) patients with hereditary diseases causing GC, such as hereditary diffuse GC, Lynch syndrome, and familial polyposis; or (3) patients receiving highly advanced medical care not covered by insurance.

Only correctly identified incident cases were included in the evaluation of accuracy in progression phase determination.

### Algorithm development and validation

This study consisted of three phases:

1. Algorithm development phase: Development and optimization of a claims-based algorithm to accurately identify and determine disease progression phases in incident cases. The targeted cohort comprised individuals who visited two large tertiary care medical centers (Hospitals A and B) from April to September 2017 and from April to September 2019 (development cohort). The cohort periods were split into 2017 and 2019 to account for changes in clinical practice with the introduction of immune checkpoint inhibitors [11, 12]. The claims dataset used for extraction of data on the targets covered the period from April 2016 to February 2021 (development dataset) to capture the claims history before cancer treatment, filter out prevalent cases, and understand the post-treatment claims history for a comprehensive assessment of the disease progression phases. An algorithm based on a combination of the International Classification of Diseases, 10th Revision (ICD-10) diagnosis codes for GC (C160-169) and claim codes for GC-specific treatment (Online Resource Table 1) was prototyped according to our previous study [26] and applied to the development dataset. Regarding some claims, the ICD-10 codes were unavailable. Therefore, we substituted the corresponding Japanese disease name codes (Online Resource Table 2). The extracted cases were matched with incident cases in the development cohort and incorrect cases were identified. Additional constraints were incorporated into the algorithm to achieve a positive predictive value (PPV) of 90% and a sensitivity of 90% to enhance its clinical acceptability. The algorithm was customized to categorize the identified cases using our previously described method [26]. The disease progression phase in each case was determined based on the combination of GC-specific treatments administered within 1.5 years of the index month. As shown in the flowchart (Online Resource Fig. 2), the patients were systematically categorized into the following three groups: endoscopic, surgical, and palliative groups.

- Endoscopic group
  –Patients for whom endoscopic resection was performed in the index month and no specific treatment other than endoscopic resection was administered thereafter.
- Surgical group
  –Patients for whom endoscopic resection was performed in the index month followed by radical surgery. These were likely to be cases in which endoscopic resection resulted in non-curative resection and additional surgery was required.
  –Patients who underwent radical surgery in the index month and received no specific treatment thereafter.
  –Patients who underwent radical surgery in the index month followed by chemotherapy. They were considered surgically curative if the guideline-recommended adjuvant chemotherapy regimens (S-1, S-1 plus oxaliplatin [SOX], or docetaxel plus oxaliplatin) [5] were initiated within 2 months post-surgery, and lasted no longer than 12 months.
  –Patients who underwent chemotherapy in the index month followed by radical surgery. They were also considered surgically curative if they were administered the guideline-recommended regimens for neoadjuvant chemotherapy (< 3 months of SOX or S-1 plus cisplatin) [5].
- Palliative group
  –Patients who underwent gastrojejunostomy, endoscopic stent placement, or radiotherapy in any month. These patients were likely to have advanced GC with gastrointestinal obstruction or metastasis.
  –Patients who underwent radical surgery in the index month and underwent chemotherapy ≥3 months after surgery. These patients were likely to have developed postoperative recurrence.
  –Patients receiving chemotherapy ≤2 months after surgery were assigned to the palliative group if the duration was 13 months or longer or if agents not included in the recommended adjuvant regimen were used.

The assignment of each case was verified against the progression phase recorded in the electronic medical records to identify inaccuracies. Adjustments were made to the algorithm constraints to ensure a higher accuracy in progression phase determination.

The algorithm was finalized in this phase and was not altered thereafter.

(2) Temporal validation phase: Validation of the developed algorithm using independent cohorts from different periods of the same institutions. The targeted cohort included patients who visited Hospitals A and B from October 2017 to March 2019 and from October 2019 to March 2021 (temporal validation cohort), and their claims datasets from October 2016 to August 2022 (temporal validation dataset) were used.
(3) External validation phase: Validation of the developed algorithm using an independent cohort from a different type of institution, i.e., a community hospital. The targeted cohort included patients who visited Hospital C between April 2017 and February 2023 (external validation cohort) and the claims dataset of Hospital C between April 2016 and July 2024 (external validation dataset).

### Outcome measures

We analyzed the patients’ baseline characteristics, including sex, age in the index month, and their progression phases after the initial series of treatments (up to 1.5 years from the index month).

The main outcome measure was the diagnostic performance of the claims-based algorithm in identifying patients with incident GC and determining their progression phases. PPV (correctly identified patients with incident GC / total patients identified as incident GC patients) and sensitivity (correctly identified patients with incident GC /total patients with incident GC) were calculated to identify patients with incident GC. Because we did not review all the electronic medical records of patients who were not diagnosed with GC in any data source, non-incident GC could not be ascertained in this study. Therefore, specificity (correctly judged as patients with non-incident GC /total patients with non-incident GC) and negative predictive value (NPV) (correctly judged as patients with non-incident GC/total patients judged as non-incident GC patients) were not presented.

Diagnostic accuracy (cases with correctly determined progression phase/total cases) was also calculated for the progression phase determination of correctly identified patients with incident GC.

Subgroup analysis was used to evaluate the variations in algorithm performance across institutions and study periods. PPV and sensitivity for identifying incident GC cases and the diagnostic accuracy for categorizing in the development cohort and temporal validation cohort were calculated separately for Hospitals A and B and for the time intervals from April to September 2017 and April to September 2019. Additionally, to investigate whether age affected the algorithm performance, these metrics were also computed for different age brackets (≤ 39, 40–49, 50–59, 60–69, 70–79, and ≥ 80 y). Given the duration covered by the claims data in this study, a 1-year washout period to exclude prevalent cases was initially selected as a practical approach, and we also investigated whether extending the washout period (1.5, 2, 2.5, and 3 years) would improve the algorithm’s performance in identifying incident GC cases, analyzing cases from April to September 2019 in the development cohort.

### Statistical analysis

The results are presented as the mean (standard deviation) for continuous variables. Categorical variables are presented as numbers and percentages, and algorithm performance metrics are presented as point estimates with 95% confidence intervals (CI). EZR version 1.27 (Saitama Medical Center, Jichi Medical University, Japan), a graphical user interface for R (The R Foundation for Statistical Computing, Vienna, Austria, version 4.1.1) was used for statistical analysis.

## Results

### Patient characteristics

We performed chart reviews of “candidate patients with incident GC”, who were identified using the cancer registry and claims information at the three participating institutions. The number of patients with incident GC was 355 (7 from only the cancer registry, 14 from only claims, and 334 from both) in the development cohort; 1142 (23 from only the cancer registry, 31 from only claims, and 1088 from both) in the temporal validation cohort; and 95 (1 from only the cancer registry, 7 from only claims, and 87 from both) in the external validation cohort (Online Resource Fig. 3). None of the patients opted out. The mean ages (SD) of patients with incident GC in the development cohort, temporal validation cohort, and external validation cohort were 72.0 (9.9), 71.2 (10.7), and 73.2 (11.8) years, respectively; the proportions of male patients were 70.4%, 69.1%, and 68.4%, respectively (Table 1). The disease progression phases in patients in each cohort are shown in Table 1. The number of patients in the endoscopic curative, surgical curative, and non-curative groups was 156, 131, and 68 in the development cohort; 493, 406, and 243 in the temporal validation cohort; and 20, 54, and 21 in the external validation cohort, respectively.

**Table 1:** Patients’ characteristics in each cohort

	Development cohort (*n* = 355)	Temporal validation cohort (*n* = 1142)	External validation cohort (*n* = 95)
Sex, male/female (% male)	250/105 (70.4%)	789/353 (69.1%)	65/30 (68.4%)
Age in years, mean (SD)	72.0 (9.9)	71.2 (10.7)	73.2 (11.8)
Progression phase			
Endoscopic curative group, n (%)	156 (43.9%)	493 (43.2%)	20 (21.1%)
Surgical curative group, n (%)	131 (36.9%)	406 (35.6%)	54 (56.8%)
Non-curative group, n (%)	68 (19.2%)	243 (21.3%)	21 (22.1%)

*SD* standard deviation

### Algorithm development

**Fig. 1:**
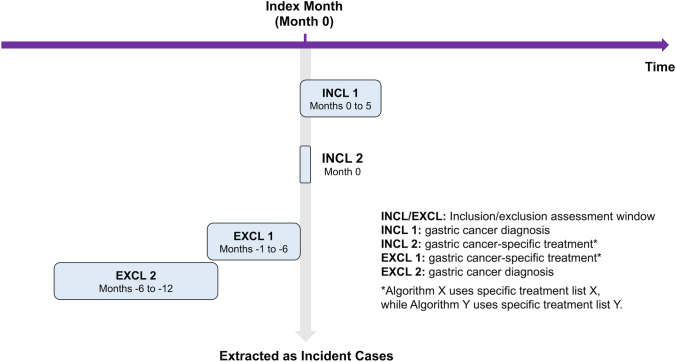
The algorithm diagram depicts how incident gastric cancer cases in a given month are identified using claims data. It is read from top to bottom, showing the sequence of actions taken for patient extraction. First, patients diagnosed with GC within 6 months including the index months are included in inclusion assessment window 1 (INCL 1). Next, patients receiving specific treatments in the index month are selected in INCL 2. Prevalent cases are filtered out in exclusion assessment windows 1 and 2 (EXCL 1 and 2). Patients receiving any listed treatments within 6 months before the index month are considered as those under ongoing treatment and excluded (EXCL 1). Additionally, patients diagnosed with GC between 12 and 6 months before the index month are excluded to eliminate recurrence (EXCL 2). These constraints result in the extraction of incident cases

**Fig. 2:**
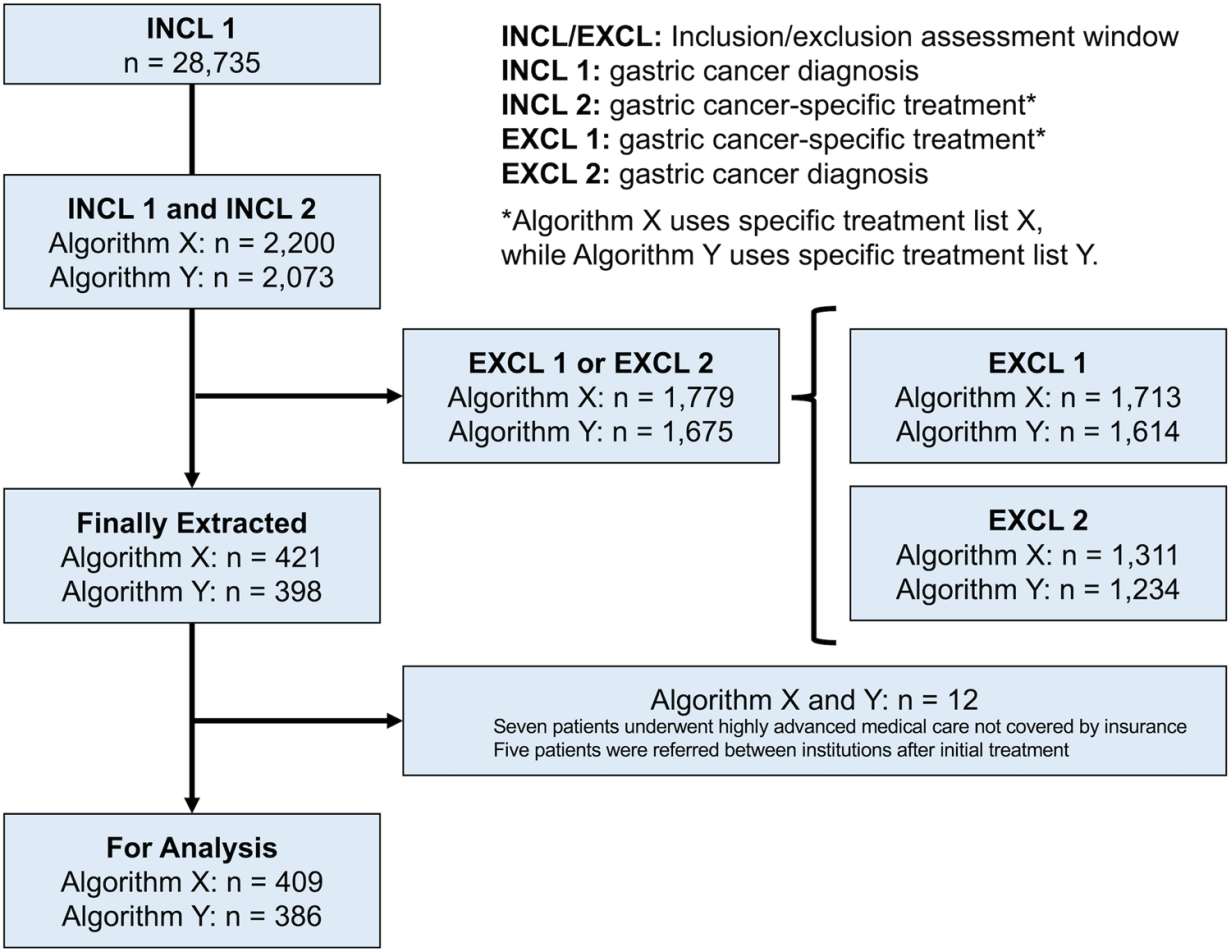
A flowchart showing the extraction of patient data by the algorithms developed in this study

As detailed in the following subheadings, we initially prototyped an algorithm to identify incident GC cases (referred to as Algorithm X) according to our previous study [26]. After evaluating the performance of Algorithm X, we refined and named it the improved version Algorithm Y. The difference between Algorithm X and Y was in the specific treatment lists used for patient extraction. Algorithm X utilized specific treatment list X, whereas Algorithm Y employed specific treatment list Y (Online Resource Table 1). Figure 1 shows a diagram depicting how Algorithm X and Y could identify incident GC cases using claims data, and Fig. 2 shows a flowchart of patient selection according to Algorithm X and Y.

#### The prototyping process of Algorithm X

Patient identification: We initially identified patients who had been diagnosed with GC (ICD-10 code C160-169 or corresponding Japanese claim codes for diagnosis) (Online Resource Table 2) within 6 months including the index months, to ensure comprehensive capture of all relevant cases. This extended inclusion period was set because we anticipated instances where the diagnosis might have been delayed due to factors such as changes in diagnosis after pathological results or significant delays in establishing the diagnosis by physicians. Subsequently, patients who received specific treatments in the index month listed in specific treatment list X (Online Resource Table 1), which constituted a comprehensive list of GC-specific surgical procedures and GC-specific chemotherapy medications in addition to endoscopic treatment, radiation therapy, and treatment of obstruction, were included. Accordingly, 2,200 cases were extracted, which was far from the true number of patients with incident GC (*n* = 355) since prevalent cases (e.g., patients under ongoing treatment and recurrent cases) were included.

Filtering prevalent cases: To ensure accuracy, we implemented constraints to exclude the prevalent cases in the index month. Patients who received any listed treatments within 6 months before the index month were considered as those under ongoing treatment and were excluded. Additionally, patients diagnosed with GC between 12 and 6 months before the index month were excluded to eliminate recurrence. These constraints resulted in the exclusion of 1,779 cases, leaving 421 cases.

Results of Algorithm X: After a chart review, 12 cases were removed, including 7 of patients undergoing highly advanced medical care not covered by insurance and 5 referred between institutions after receiving initial GC-specific treatment, resulting in 409 cases for analysis. Performance metrics showed a PPV and sensitivity of 85.3% (95% CI, 81.5–88.6%) (349/409) and 98.3% (95% CI, 96.4–99.4%) (349/355), respectively. This algorithm yielded 60 false positives, which were categorized into four types: (1) non-epithelial tumors labeled as GC and treated with surgery or other procedures (41.7%, 25/60), (2) gastric adenomas labeled as GC and treated with endoscopic treatments (33.3%, 20/60), (3) cases labeled as GC and treated with chemotherapy or radiotherapy for other organ cancers (21.7%, 13/60), and (4) recurrent GC (3.3%, 2/60).

#### Algorithm refinement

Algorithm Y incorporated an updated specific treatment list (specific treatment list Y), excluding surgeries rarely performed for GC and drugs not recommended in the guidelines (Online Resource Table 1). The refined algorithm extracted 398 cases. After a chart review, the same 12 cases that were excluded from Algorithm X analysis were removed, resulting in 386 cases for analysis. Algorithm Y demonstrated improved accuracy with a higher PPV (90.2%, 95% CI, 86.7–92.9%, 348/386) (Table 2). The 38 false positives included non-epithelial tumors (23.7%, 9/38), gastric adenomas treated with endoscopic treatments (52.6%, 20/38), cases of GC diagnosis treated for other organ cancers (18.4%, 7/38), and recurrent GC (5.3%. 2/38). When gastric adenomas were included in the definition of GC, the PPV of Algorithm Y was 95.3% (95% CI, 92.7–97.2%) (368/386). Algorithm Y outperformed Algorithm X and was adopted as the established algorithm for identifying incident GC cases in this study.

**Table 2:** Performance metrics of Algorithm Y in the identification of incident gastric cancer cases in each cohort

	PPV [% (95% CI)]	SEN [% (95% CI)]
Development cohort (*n* = 355)	90.2% (86.7–92.9%)	98.0% (96.0–99.2%)
	(348/386)	(348/355)
Temporal validation cohort (*n* = 1142)	90.0% (88.1–91.6%)	98.0% (97.0–98.7%)
	(1119/1244)	(1119/1142)
External validation cohort (*n* = 95)	95.9% (89.9–98.9%)	98.9% (94.3–100%)
	(94/98)	(94/95)

*PPV* positive predictive value, *SEN* sensitivity, *CI* confidence interval

#### Progression phase determination

**Fig. 3:**
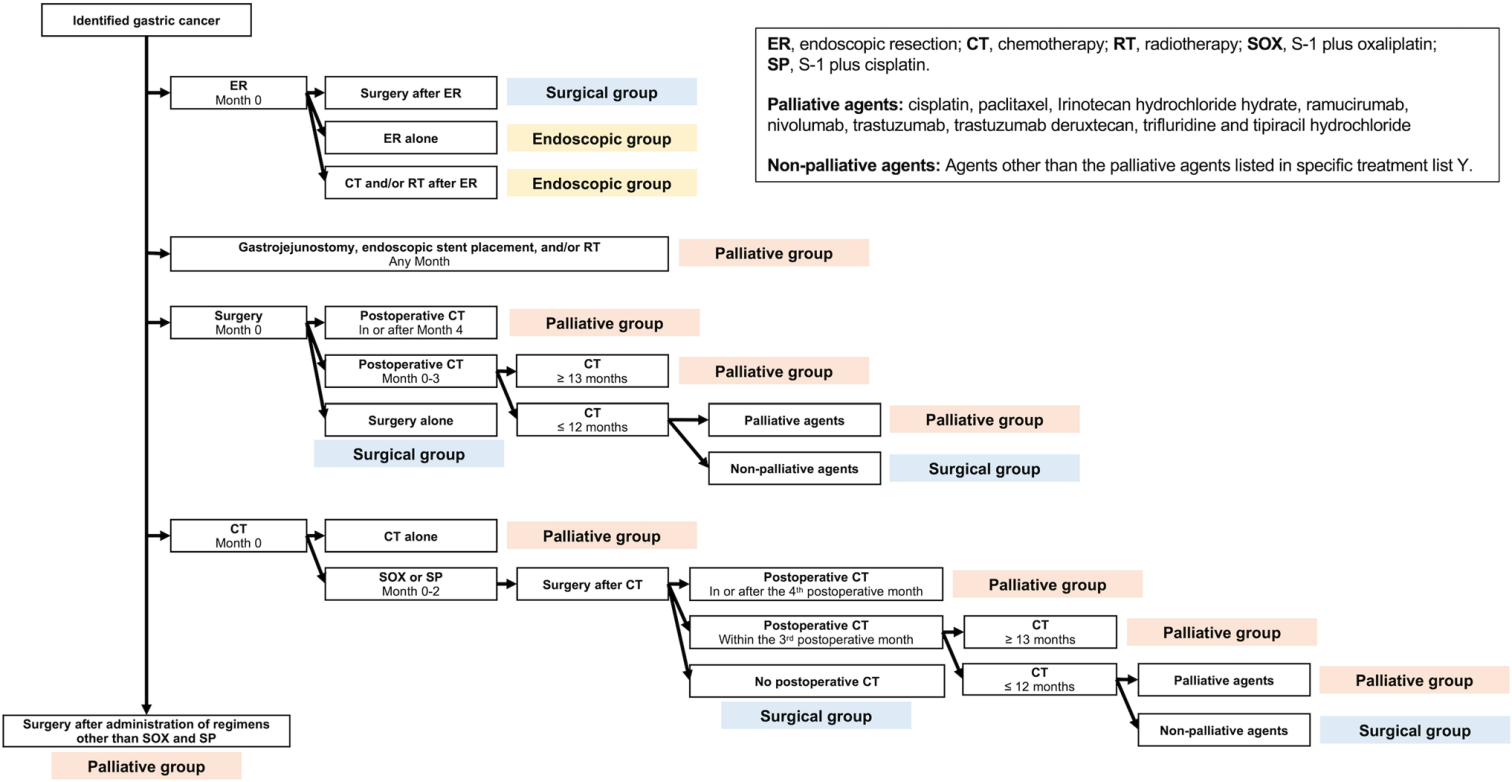
A refined algorithm for progression phase determination based on the initial series of treatments

Based on the aforementioned methodology, Algorithm Y was customized to determine the disease progression phase of the identified patients with incident GC. The initial diagnostic accuracy of progression phase determination was 92.8% (95% CI, 89.6–95.3%) (323/348). Additional constraints were integrated for refinement (Fig. 3).

- Endoscopic group
  –Patients who initially underwent endoscopic treatment in the index month, followed by chemotherapy or radiotherapy. In these cases, early GC was cured by endoscopic resection and chemotherapy and radiotherapy were likely administered for concurrent cancers of other organs.

Moreover, given individual variability in the postoperative recovery time, the period for chemotherapy initiated after radical surgery, i.e., postoperative chemotherapy, changed from within 2 months to within 3 months postoperatively.

These adjustments improved the diagnostic accuracy of progression phase determination to 94.5% (95% CI, 91.6–96.7%) (329/348) (Table 3).

**Table 3:** Diagnostic accuracy of the developed algorithm in progression phase determination in each cohort

	Diagnostic accuracy [% (95% CI)]
Development cohort (*n* = 348)	94.5% (91.6–96.7%)
	(329/348)
Temporal validation cohort (*n* = 1119)	94.1% (92.6–95.4%)
	(1053/1119)
External validation cohort (*n* = 94)	93.6% (86.6–97.6%)
	(88/94)

*CI* confidence interval

### Validation of the developed algorithm

The developed algorithm was applied to the temporal validation dataset to evaluate its performance on the temporal validation cohort. PPV and sensitivity in identifying patients with incident GC were 90.0% (95% CI, 88.1–91.6%) (1119/1244) and 98.0% (95% CI, 97.0–98.7%) (1119/1142), respectively (Table 2). The diagnostic accuracy for progression phase determination was 94.1% (95% CI, 92.6–95.4%) (1053/1119) (Table 3).

Similarly, the algorithm was applied to the external validation dataset to evaluate its performance on the external validation cohort. PPV and sensitivity in identifying patients with incident GC were 95.9% (95% CI, 89.9–98.9%) (94/98) and 98.9% (95% CI, 94.3–100.0%) (94/95), respectively (Table 2). The diagnostic accuracy in progression phase determination was 93.6% (95% CI, 86.6–97.6%) (88/94) (Table 3).

### Subgroup analysis

Subgroup analysis showed no apparent differences in algorithm performance between the institutions and study periods (Online Resource Tables 3 and 4). Online Resource Tables 5 and 6 present the differences in algorithm performance across age brackets. The algorithm’s accuracy for progression phase determination was the lowest at 90.5% (95% CI, 82.1–95.8%) (76/84) in the development cohort for those aged ≥ 80 years.

### Differences in the algorithm performance by washout periods

Online Resource Table 7 shows the performance differences in the identification of incident GC cases when the washout period varied from 1 year to 1.5, 2, 2.5, and 3 years, using the development cohort from April to September 2019. When the washout period was set to 1 year, the sensitivity was 98.3% (95% CI, 95.1–99.6%) (174/177) and the PPV was 89.7% (95% CI, 84.5–93.6%) (174/194), whereas when the washout period was extended to 3 years, the sensitivity was 97.7% (95% CI, 94.3–99.4%) (173/177) and the PPV was 90.1% (95% CI, 85.0–93.9%) (173/192). The number of extracted cases and algorithm performance were almost identical across different washout periods.

## Discussion

In the present study, we developed a claims-based algorithm for identifying and determining the progression phases of incident GC cases in Japan using a claims database of two large tertiary care medical centers and validated the algorithm’s performance using different types of datasets. Achieving a PPV and sensitivity of 90% for the identification of incident GC cases, the algorithm represents a significant improvement over the existing methods. Furthermore, the diagnostic accuracy of progression phase determination exceeded 90%. To the best of our knowledge, this is the first validation study of claims-based algorithms for determining the disease progression phase in patients with incident GC.

High PPV and high sensitivity of our claims-based algorithm for identifying incident GC were achieved by incorporating real-world and clinical expert insights from board-certified gastroenterologists during the development process. We developed a list of specific treatment codes based on the GC guidelines and implemented a sufficient washout period to exclude prevalent cases, reflecting actual patient visit intervals in clinical practice. This meticulous approach has led to a more clinically relevant and accurate algorithm than previous methods. Some prior studies have made significant attempts at similar algorithm development, but with limitations. For example, the algorithm of de Luise et al. showed a PPV of 76.83% and a sensitivity of 22.11%, which was likely hindered by the omission of endoscopic treatment codes [32]. Similarly, Ihira et al. and Ogawa et al. contributed valuable findings but faced challenges, such as the applicability of their algorithms across all Japanese medical institutions and the use of anonymized data that precluded verification with actual medical records or cancer registries [31, 33]. In this study, we were unable to assess the specificity and NPV in identifying patients with incident GC. However, considering that the vast majority of patients in each cohort were not those with GC assuming that both would be close to 100% is reasonable, similar to that in previous reports [31, 32]. Moreover, unlike previous studies, our study uniquely incorporated the assessment of GC progression phases into the algorithm. Based on our prior experience with colorectal cancer staging [26], we applied similar logic to enhance the determination of GC progression phases, achieving an accuracy approaching 95%.

These advancements facilitated the precise identification of patients with incident GC and determining their disease progression phases from the claims databases. This algorithm is particularly valuable for understanding treatment patterns and outcomes in real-world clinical practice that cannot be examined in clinical trials. Additionally, this algorithm can contribute to the analysis of progression phase-specific medical costs of real-world patients with GC [26]. The claims data used in this study were maintained independently by each institution; cases that were transferred to or from other facilities were excluded from the analysis due to incomplete clinical records. However, such cases were few (1.3% or 5 of 398 cases extracted by Algorithm Y in the algorithm development phase); their exclusion would have had minimal impact on the study’s findings. The results of this study could be applied to the health insurance claims databases held by each insurer, in which each patient’s complete medical history is listed. Furthermore, since cancers in other organs may be treated with the same chemotherapeutic agents used for GC, there is a risk of overestimating the progression phase of GC in cases of concurrent cancers of other organs. This issue was more evident in large tertiary care medical centers, which have more complicated cases but was less significant in community hospitals. Consequently, the influence of this factor is minimal when employing this algorithm across the health insurer-based claims database and National Database, which represent the entire Japanese healthcare landscape.

Despite its strengths, this study has some limitations. First, approximately half of the false-positive cases were gastric adenomas, suggesting that there may be a mixture of diseases with low malignant potential among the cases determined to be endoscopically curative. However, in Japan, such tumors are often treated in the same manner as early GC, and this type of misclassification is not considered a major problem in clinical practice. If gastric adenomas were included in the definition of GC in the algorithm development phase, the PPV of Algorithm Y would improve from 90.2% (95% CI, 86.7–92.9%) (348/386) to 95.3% (95% CI, 92.7–97.2%) (368/386). Second, this algorithm identified patients with GC using the claims code of GC-specific treatment, making it impossible to identify patients with diagnosed GC who have been followed up without treatment or those who have been provided the best supportive care from the time of diagnosis owing to advanced age or comorbidities. To broadly identify such cases, future studies may be required to develop an algorithm that incorporates constraints that consider the patients’ general conditions and comorbidities. Third, the performance of the algorithm varied across age groups. Particularly in older patients, some of whom cannot tolerate chemotherapy, when surgery alone is performed without chemotherapy, the incurable progression phase of the patient’s disease may be underestimated because the algorithm determines that the patient is in the surgical curative group. Introducing constraints that adjust the progression phase assessment based on age and treatment combinations may help provide more accurate categorizing of older patients. Fourth, this algorithm determines the progression phase after the initial series of treatments (up to 1.5 years from the index month), which means that it does not strictly assess the initial progression phase at diagnosis but rather includes progression phase that may have reflected short-term recurrence. However, it can be argued that patients with rapid disease recurrence after initial treatment likely have high malignant potential from the outset. Thus, the algorithm might reflect the intrinsic malignancy of cancer. Fifth, the external validation cohort was derived from a single institution and the sample size remains limited. However, PPV was significantly higher in the external validation cohort than that in the temporal validation cohort, with a lower limit of the 95% CI at 89.9%, which is clinically acceptable. To further enhance the reliability of our study results, additional external validation using larger datasets from multiple institutions is warranted.

Regarding generalizability, although the algorithm works well in Japan, different healthcare systems and coding practices may require adjustments before applying this model internationally. In addition, this algorithm was developed based on the current GC treatment guidelines and may need to be adjusted in future with changes in clinical practices and coding lists. Furthermore, with the introduction of new drugs, continually customizing the algorithm to maintain its accuracy will be necessary.

In conclusion, this study presents a robust, validated, claims-based algorithm for identifying and determining the progression phases of incident GC cases in Japan. This algorithm can facilitate retrospective studies and population-based analyses, shedding light on the economic and physical burden of GC, and providing targeted prevention and treatment strategies in future studies, leading to the improvement in real-world clinical outcomes of patients with GC.

## Supplementary Information

Below is the link to the electronic supplementary material.

Supplementary file1 (TIFF 27929 kb)

Supplementary file2 (TIFF 19784 kb)

Supplementary file3 (TIFF 28960 kb)

Supplementary file4 (DOCX 39 kb)
