# Supplementary figures and images for: Development and validation of a claims-based algorithm to identify incidents and determine the progression phases of gastric cancer cases in Japan
Source: J Gastroenterol. 2024 Nov 26;60(2):141–51. doi: 10.1007/s00535-024-02167-y (PMC11794417; doi:10.1007/s00535-024-02167-y)

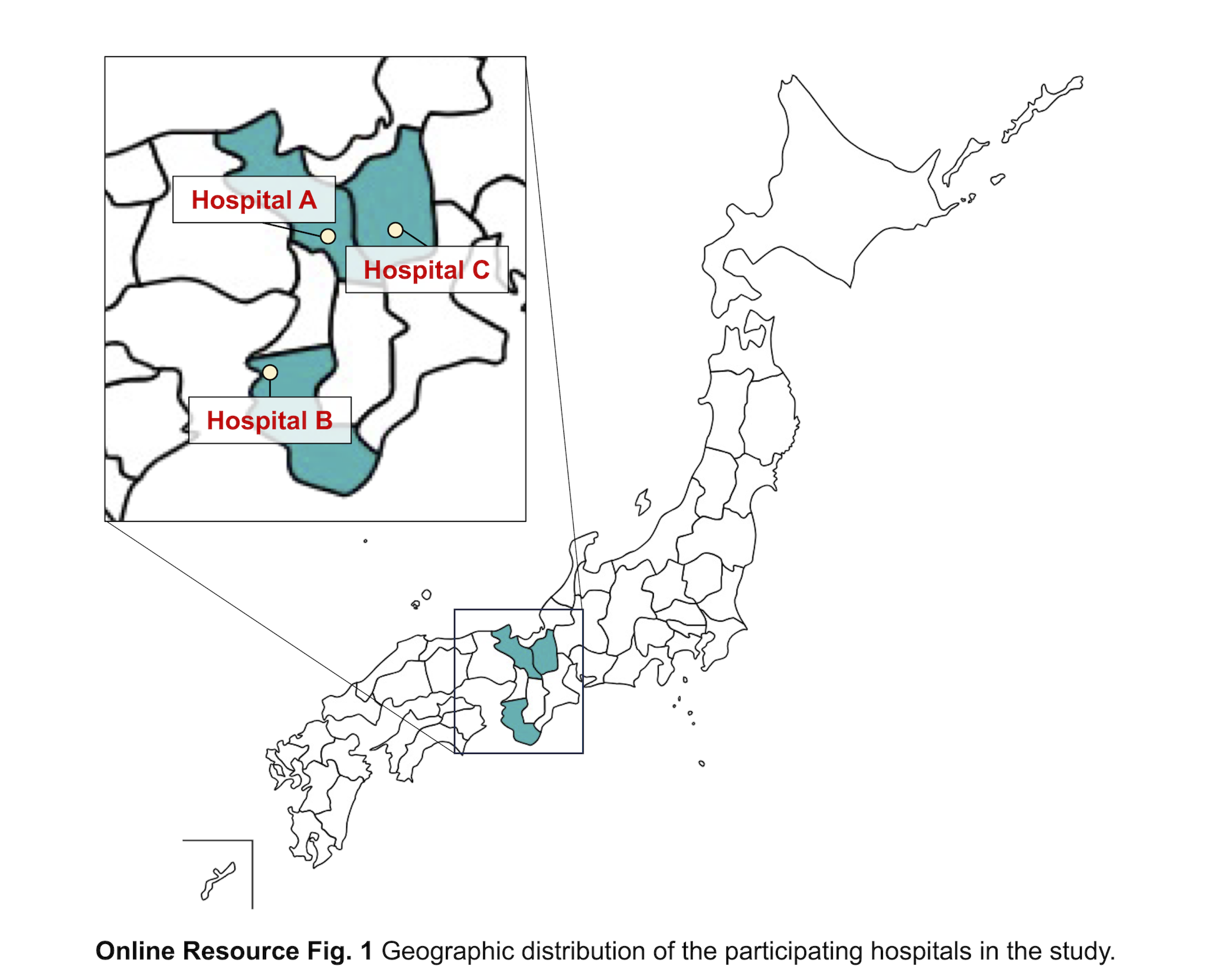

Supplement: Supplementary file 1 — Supplementary file1 (TIFF 27929 kb) [file 535_2024_2167_MOESM1_ESM.tiff]

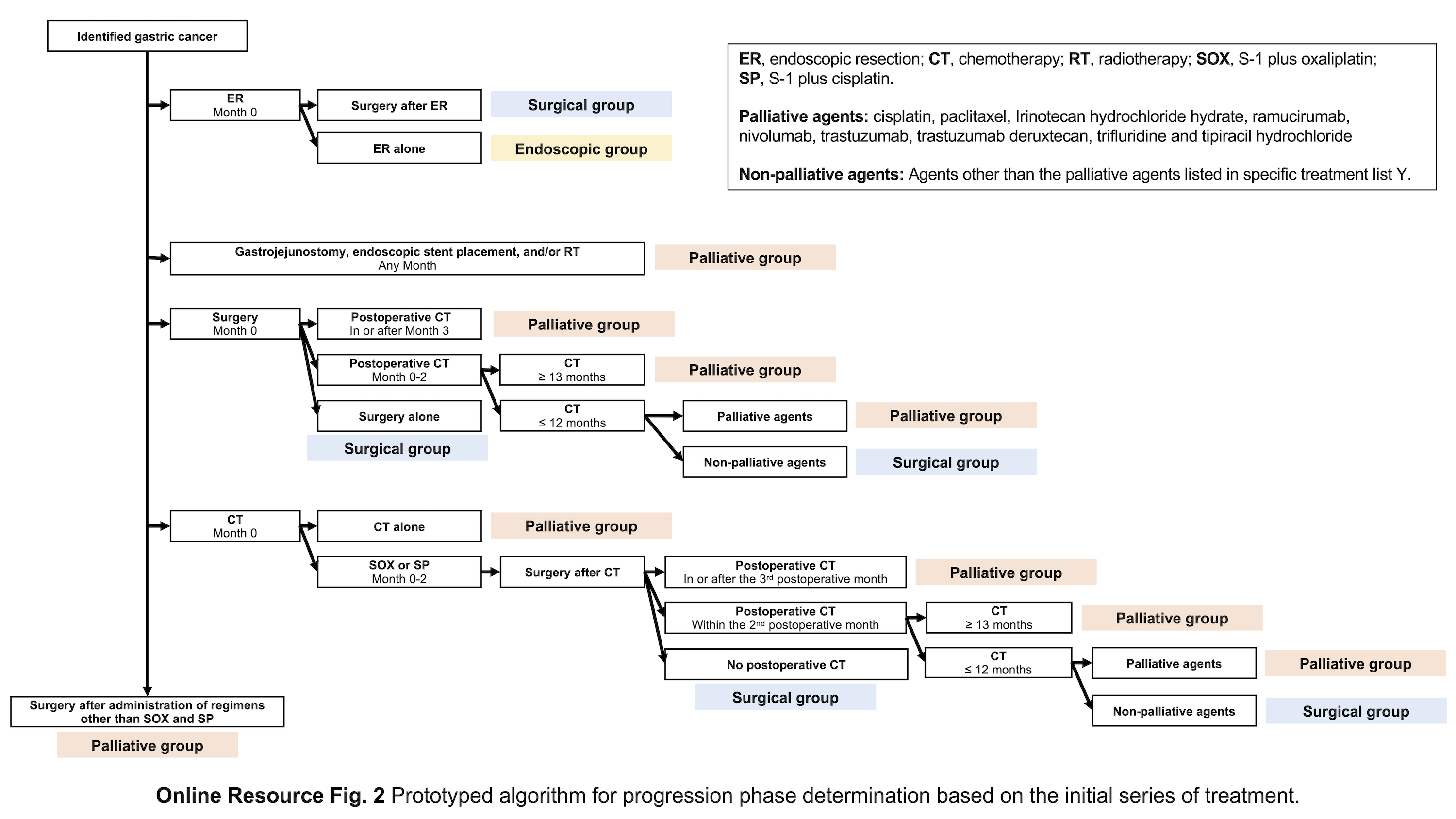

Supplement: Supplementary file 2 — Supplementary file2 (TIFF 19784 kb) [file 535_2024_2167_MOESM2_ESM.tiff]

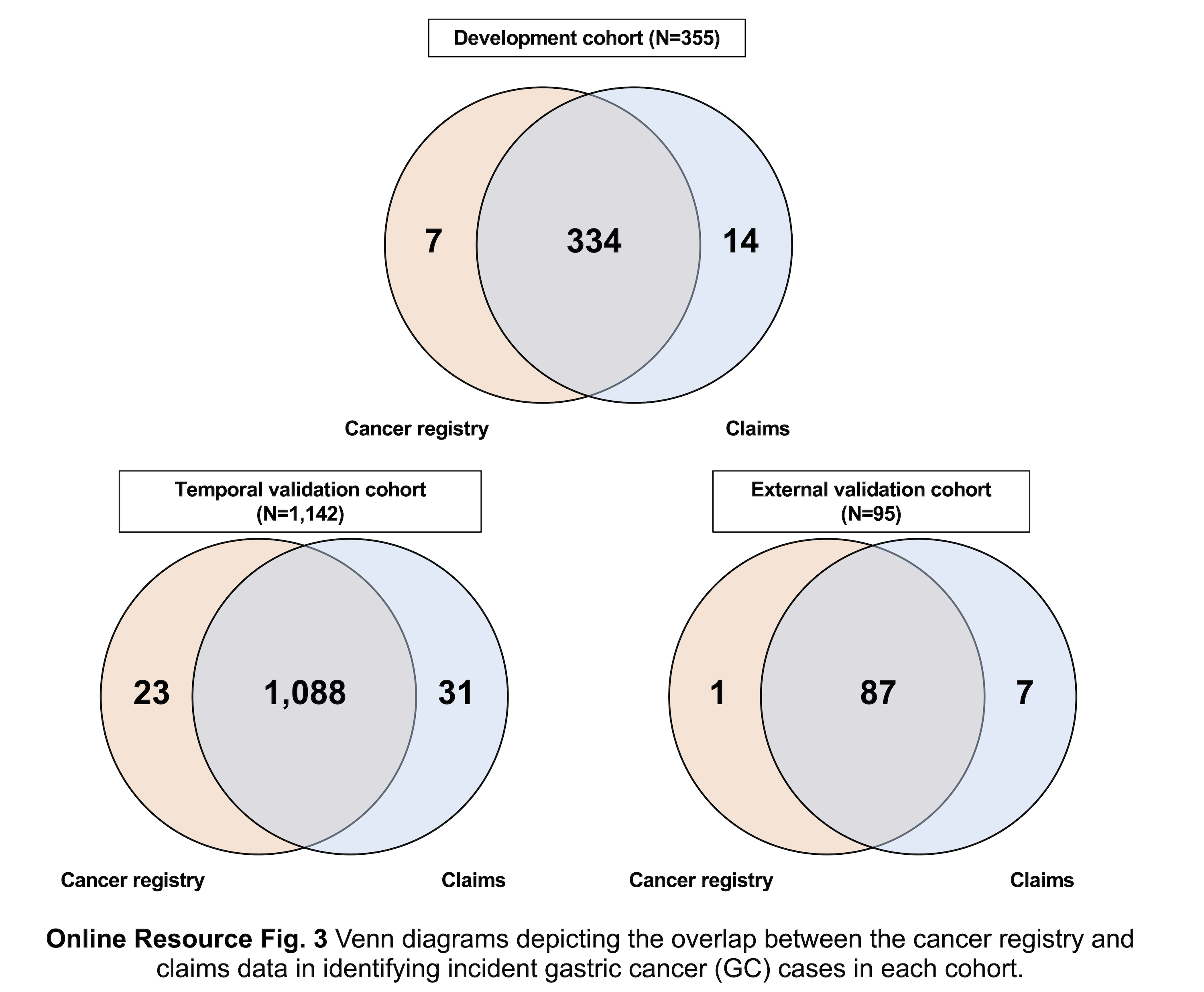

Supplement: Supplementary file 3 — Supplementary file3 (TIFF 28960 kb) [file 535_2024_2167_MOESM3_ESM.tiff]
