# Supplementary material for: Development and validation of a claims-based algorithm to identify incidents and determine the progression phases of gastric cancer cases in Japan
Source: J Gastroenterol. 2024 Nov 26;60(2):141–51. doi: 10.1007/s00535-024-02167-y (PMC11794417; doi:10.1007/s00535-024-02167-y)
Supplement: Supplementary file 4 — Supplementary file4 (DOCX 39 kb) [file 535_2024_2167_MOESM4_ESM.docx]

**Development and validation of a claims-based algorithm to identify incidents and determine the progression phases of gastric cancer cases in Japan**

**Journal name: *The Journal of Gastroenterology***

Takahiro Inoue^1^*, Nobukazu Agatsuma^1,2,3^*, Takahiro Utsumi^1^, Yukari Tanaka^1^, Yoshitaka Nishikawa^4,5^, Takahiro Horimatsu^6^, Takahiro Shimizu^1^, Mitsuhiro Nikaido^1^, Yuki Nakanishi^1^, Nobuaki Hoshino^7^, Yoshimitsu Takahashi^4^, Takeo Nakayama^4^, and Hiroshi Seno^1^

*Contributed equally

^1^Department of Gastroenterology and Hepatology, Kyoto University Graduate School of Medicine, 54 Kawaharacho, Shogoin, Sakyo-ku, Kyoto 606-8507, Japan

^2^Department of Gastroenterology and Hepatology Japanese Red Cross Wakayama Medical Center Wakayama Japan.

^3^Department of Internal Medicine, Hino Memorial Hospital, Shiga, Japan.

^4^Department of Health Informatics, Kyoto University School of Public Health, Kyoto, Japan

^5^Department of Clinical Oncology, Kyoto University Hospital, Kyoto, Japan

^6^Institute for Advancement of Clinical and Translational Science (iACT), Kyoto University Hospital, Kyoto, Japan

^7^Department of Surgery, Kyoto University Graduate School of Medicine, Kyoto, Japan

**Correspondence**

Takahiro Utsumi MD, PhD

Department of Gastroenterology and Hepatology, Kyoto University Graduate School of Medicine, 54 Kawaharacho, Shogoin, Sakyo-ku, Kyoto 606-8507, Japan

Tel: 81-75-751-4319; Fax: 81-75-751-4303; E-mail: tk_utsumi@kuhp.kyoto-u.ac.jp

**Supplementary Materials**

**Online Resource Fig. 1** Geographic distribution of the participating hospitals in the study.

Hospital A: Kyoto University Hospital

A large tertiary care medical center with 1121 beds, serving as part of the development and the temporal validation cohort.

Hospital B: Japanese Red Cross Wakayama Medical Center

Another large tertiary care medical center with 700 beds also served as part of the development and the temporal validation cohort.

Hospital C: Hino Memorial Hospital

A community hospital with 150 beds, included as the external validation cohort.

**Online Resource Fig. 2** Prototyped algorithm for progression phase determination based on the initial series of treatment.

**Online Resource Fig. 3** Venn diagrams depicting the overlap between the cancer registry and claims data in identifying incident gastric cancer (GC) cases in each cohort.

**Online Resource Table 1** The codes associated with gastric cancer (GC)-specific procedures, and the claim computer processing system codes for the anticancer agents used for GC treatment

|  | Specific treatment list X | Specific treatment list Y |
| --- | --- | --- |
|  | Procedure code | |
| **Endoscopic treatment** | | |
| Polypectomy or endoscopic mucosal resection or endoscopic submucosal dissection | 150276310, 150276410, 150323010, 150164410 | 150276310, 150276410, 150323010, 150164410 |
| **Surgical treatment with resection of primary lesion** | | |
| Surgery with gastrectomy | 150168010, 150168110, 150337310, 150429010, 150165210, 150166110, 150337210 | 150168010, 150168110, 150337310, 150429010 |
| Laparoscopic surgery with gastrectomy | 150323510, 150323710, 150377910, 150406710, 150406910, 150407110, 150429110, 150323410, 150323610, 150377810, 150406610, 150406810, 150407010 | 150323510, 150323710, 150377910, 150406710, 150406910, 150407110, 150429110 |
| Local resection of primary lesion | 150164210, 150323210, 150377610, 150377710 | 150164210 |
| **Surgical treatment without resection of primary lesion** | 150162910, 150163010, 150171210, 150271650 | – |
| **Radiotherapy** | | |
| X-ray therapy | 180008810, 180019410 | 180008810, 180019410 |
| High-energy radiotherapy | 180020710, 180020810, 180020910, 180021010, 180021110, 180021210, 180021310, 180021410, 180021510, 180021610, 180021710, 180021810, 180021910, 180022010 | 180020710, 180020810, 180020910, 180021010, 180021110, 180021210, 180021310, 180021410, 180021510, 180021610, 180021710, 180021810, 180021910, 180022010 |
| **Treatment for obstruction** | | |
| Gastrojejunostomy | 150171310, 150362010 | 150171310, 150362010 |
| Stent placement | 150281350, 150347610 | 150281350, 150347610 |
| **Chemotherapy (Generic name)** | Claim computer processing system code | |
| Irinotecan hydrochloride hydrate | 620007257, 620007258, 620009516, 620009520, 620009515, 620009519, 620009518, 620009522, 620919501, 620919701, 622019401, 622019501, 622059701, 622059801, 622258901, 622259001, 622236901, 622237001, 622230201, 622230301, 622470401, 622470501, 621900302, 621900402, 622903700, 622903800, 622091101, 622091201 | 620007257, 620007258, 620009516, 620009520, 620009515, 620009519, 620009518, 620009522, 620919501, 620919701, 622019401, 622019501, 622059701, 622059801, 622258901, 622259001, 622236901, 622237001, 622230201, 622230301, 622470401, 622470501, 621900302, 621900402, 622903700, 622903800, 622091101, 622091201 |
| Tegafur, Uracil | 620915001, 621929901, 621930001, 621930101 | 620915001, 621929901, 621930001, 621930101 |
| Oxaliplatin | 621932201, 621932301, 622189401, 622374801, 622374901, 622371801, 622371901, 622411901, 622371101, 622371201, 622426801, 622373201, 622373301, 622414601, 622385701, 622385801, 622434901, 622437201, 622437301, 622437401, 622389801, 622389901, 622431101, 622388601, 622388701, 622428001, 622393201, 622393301, 622437001, 622460601, 622394701, 622394801, 622432401, 622392001, 622392101, 622439101, 622383201, 622383301, 622461701, 622394703, 622394803, 622432403, 622617800, 622476900, 622617900, 622381301, 622381401, 622417801 | 621932201, 621932301, 622189401, 622374801, 622374901, 622371801, 622371901, 622411901, 622371101, 622371201, 622426801, 622373201, 622373301, 622414601, 622385701, 622385801, 622434901, 622437201, 622437301, 622437401, 622389801, 622389901, 622431101, 622388601, 622388701, 622428001, 622393201, 622393301, 622437001, 622460601, 622394701, 622394801, 622432401, 622392001, 622392101, 622439101, 622383201, 622383301, 622461701, 622394703, 622394803, 622432403, 622617800, 622476900, 622617900, 622381301, 622381401, 622417801 |
| Tegafur, Gimeracil, Oteracil potassium | 620915501, 620915601, 620009353, 620009354, 622256001, 622256101, 622254901, 622255001, 622243001, 622243101, 622275701, 622275801, 622266701, 622266801, 622294601, 622294701, 622285701, 622285801, 622397301, 622397401, 622397101, 622397201, 622434701, 622434801, 622430801, 622430901, 622487301, 622487401, 622497901, 622498001, 622537501, 622537601, 622898700, 622898800, 622898900, 622899000 | 620915501, 620915601, 620009353, 620009354, 622256001, 622256101, 622254901, 622255001, 622243001, 622243101, 622275701, 622275801, 622266701, 622266801, 622294601, 622294701, 622285701, 622285801, 622397301, 622397401, 622397101, 622397201, 622434701, 622434801, 622430801, 622430901, 622487301, 622487401, 622497901, 622498001, 622537501, 622537601, 622898700, 622898800, 622898900, 622899000 |
| Capecitabine | 610470009, 622656401, 622677701, 622674301, 622679001, 622700101, 622695801 | 610470009, 622656401, 622677701, 622674301, 622679001, 622700101, 622695801 |
| Trifluridine and tipiracil hydrochloride | 622336001, 622336101 | 622336001, 622336101 |
| Tegafur | 620004566, 620004748, 644210046, 620004820, 620910101, 620005087, 610461179 | 620004566, 620004748, 644210046, 620004820, 620910101, 620005087, 610461179 |
| Fluorouracil | 614210004, 614210003, 622229101, 622047901, 622412501, 622412601, 610461237 | 614210004, 614210003, 622229101, 622047901, 622412501, 622412601, 610461237 |
| Ramucirumab | 622417901, 622418001 | 622417901, 622418001 |
| Calcium Levofolinate hydrate | 620005725, 620005730, 620005722, 620005729, 620005717, 620005728, 620005718, 620005880, 620005720, 620005881, 620005716, 620005879, 620005719, 620008234, 620005723, 620005726, 620008543, 620005721, 620009589, 620005724, 620009590, 620007161, 620007162, 620005715, 620005727, 621813503, 622119703, 621813603, 621782504, 621897904, 622693500, 622758000, 622758100 | 620005725, 620005730, 620005722, 620005729, 620005717, 620005728, 620005718, 620005880, 620005720, 620005881, 620005716, 620005879, 620005719, 620008234, 620005723, 620005726, 620008543, 620005721, 620009589, 620005724, 620009590, 620007161, 620007162, 620005715, 620005727, 621813503, 622119703, 621813603, 621782504, 621897904, 622693500, 622758000, 622758100 |
| Cisplatin | 620004129, 620004130, 620004131, 620006298, 620006299, 620006300, 620008946, 620008947, 620008948, 620923301, 620923701, 620924101, 620923202, 620923602, 620924002, 622760800, 622760900, 622761000 | 620004129, 620004130, 620004131, 620006298, 620006299, 620006300, 620008946, 620008947, 620008948, 620923301, 620923701, 620924101, 620923202, 620923602, 620924002, 622760800, 622760900, 622761000 |
| Docetaxel hydrate | 620919901, 620919801, 622068501, 622068601, 622215301, 622215401, 622283101, 622283201, 622295501, 622295601, 622285201, 622285301, 622285401, 622272001, 622272101, 622290401, 622290501, 622294901, 622295001, 622356401, 622356501, 622354801, 622354901, 622429301, 622429401, 622435002, 622435102, 622408501, 622408601, 622231801, 622231901, 622417601, 622417701 | 620919901, 620919801, 622068501, 622068601, 622215301, 622215401, 622283101, 622283201, 622295501, 622295601, 622285201, 622285301, 622285401, 622272001, 622272101, 622290401, 622290501, 622294901, 622295001, 622356401, 622356501, 622354801, 622354901, 622429301, 622429401, 622435002, 622435102, 622408501, 622408601, 622231801, 622231901, 622417601, 622417701 |
| Trastuzumab | 622069801, 622069901, 622628901, 622629001, 622630701, 622630801, 622659701, 622659801, 622679201, 622679301, 629921901, 629922001 | 622069801, 622069901, 622628901, 622629001, 622630701, 622630801, 622659701, 622659801, 622679201, 622679301, 629921901, 629922001 |
| Trastuzumab Deruxtecan | 629907101 | 629907101 |
| Paclitaxel | 620003751, 620003752, 620004170, 620004171, 620005688, 620005689, 620005690, 622082001, 622082101, 622259101, 622259201, 622375001, 622375101, 622760500, 622760600, 622760700, 621970101, 622009102, 622009202 | 620003751, 620003752, 620004170, 620004171, 620005688, 620005689, 620005690, 622082001, 622082101, 622259101, 622259201, 622375001, 622375101, 622760500, 622760600, 622760700, 621970101, 622009102, 622009202 |
| Nivolumab | 622364801, 622364901, 629911501, 622662201 | 622364801, 622364901, 629911501, 622662201 |
| Aclarubicin hydrochloride | 620005148 | – |
| Epirubicin hydrochloride | 622760200, 620009523, 621966401, 620009526, 621966601, 622760300, 620007224, 620008174, 622760400, 620008175, 620007225, 620003790, 620003791, 620003792, 620003793, 621966701, 621966501, 622246601, 622246701 | – |
| Cyclophosphamide hydrate | 640453101, 644210037, 620005941, 622181601 | – |
| Cytarabine | 620003714, 620003715, 620003716, 620003717, 620003718 | – |
| PICIBANIL | 620004740, 620004741, 620004742, 620004743 | – |
| Doxifluridine | 614210128, 614210129 | – |
| Doxorubicin hydrochloride | 621995301, 621995401, 621983201, 621983301, 620003675, 622014001 | – |
| Pirarubicin | 620003762, 620003763, 620005206, 620005207, 622513101 | – |
| Nimustine hydrochloride | 644210020, 644210021 | – |
| Mitomycin C | 620000328, 620000329 | – |
| Methotrexate | 620002151, 620004084, 622581501, 610432016, 620004082, 620004083, 621642203, 621734801, 622742200, 622869601, 622634501, 622630501, 622841001, 621622602, 622742300, 622642401, 614210098, 620007515, 622221301, 644210049, 644210048 | – |
| Pembrolizumab | 622515801, 622515701 | – |

**Online Resource Table 2** ICD-10 codes and corresponding Japanese claim codes for gastric cancer

| ICD-10 code | Japanese claim code |
| --- | --- |
| C160 | 1510005 |
|  | 8849679 |
| C161 | 1513002 |
| C162 | 1514002 |
| C163 | 1512002 |
|  | 8848024 |
| C164 | 1511003 |
|  | 8830612 |
| C165 | 8845849 |
| C166 | 8845852 |
| C169 | 1519006 |
|  | 1519010 |
|  | 1519011 |
|  | 1519012 |
|  | 1519015 |
|  | 1519017 |
|  | 1519020 |
|  | 1519022 |
|  | 8830411 |
|  | 8830421 |
|  | 8842111 |
|  | 8842666 |
|  | 8844932 |
|  | 8846240 |
|  | 8846354 |
|  | 8847306 |
|  | 8847307 |
|  | 8847827 |
|  | 8847839 |
|  | 8849064 |
|  | 8849065 |
|  | 8849698 |
|  | 8850380 |

**Online Resource Table 3** Subgroup analysis comparing differences in the performance metrics of the algorithm in the identification of incident gastric cancer cases between facilities and study periods

|  | PPV [% (95 % CI)] | SEN [% (95 % CI)] |
| --- | --- | --- |
| Development cohort (n = 355) |  |  |
| Institution |  |  |
| Hospital A (n=101) | 92.5% (85.7–96.7%)  (98/106) | 97.0% (91.6–99.4%)  (98/101) |
| Hospital B (n=254) | 89.3% (85.1–92.7%)  (250/280) | 98.4% (96.0–99.6%)  (250/254) |
| Study period |  |  |
| April 2017 to September 2017 (n=178) | 90.6% (85.6–94.3%)  (174/192) | 97.8% (94.3–99.4%)  (174/178) |
| April 2019 to September 2019 (n=177) | 89.7% (84.5–93.6%)  (174/194) | 98.3% (95.1–99.6%)  (174/177) |
| Temporal validation cohort (n = 1142) |  |  |
| Institution |  |  |
| Hospital A (n=379) | 92.3% (89.2–94.7%)  (371/402) | 97.9% (95.9–99.1%)  (371/379) |
| Hospital B (n=763) | 88.8% (86.5–90.9%)  (748/842) | 98.0% (96.8–98.9%)  (748/763) |
| Study period |  |  |
| October 2017 to March 2019 (n=625) | 90.6% (88.2–92.7%)  (618/682) | 98.9% (97.7–99.5%)  (618/625) |
| October 2019 to March 2021 (n=517) | 89.1% (86.3–91.6%)  (501/562) | 96.9% (95.0–98.2%)  (501/517) |

PPV, positive predictive value; SEN, sensitivity; CI, confidence interval.

**Online Resource Table 4** Subgroup analysis comparing the differences in the diagnostic accuracy of the algorithm in progression phase determination between facilities and study periods

|  | Diagnostic accuracy [% (95 % CI)] |
| --- | --- |
| Development cohort (n=348) |  |
| Institution |  |
| Hospital A (n=98) | 93.9% (87.1–97.7%)  (92/98) |
| Hospital B (n=250) | 94.8% (91.3–97.2%)  (237/250) |
| Study period |  |
| April 2017 to September 2017 (n=174) | 96.0% (91.9–98.4%)  (167/174) |
| April 2019 to September 2019 (n=174) | 93.1% (88.3–96.4%)  (162/174) |
| Temporal validation cohort (n=1119) |  |
| Institution |  |
| Hospital A (n=371) | 93.8% (90.8–96.0%)  (348/371) |
| Hospital B (n=748) | 94.3% (92.3–95.8%)  (705/748) |
| Study period |  |
| October 2017 to March 2019 (n=618) | 94.3% (92.2–96.0%)  (583/618) |
| October 2019 to March 2021 (n=501) | 93.8% (91.3–95.8%)  (470/501) |

CI, confidence interval.

**Online Resource Table 5** Differences in the performance metrics of the algorithm for the identification of incident gastric cancer cases across age brackets in the development cohort.

| Age brackets | PPV [% (95 % CI)] | SEN [% (95 % CI)] |
| --- | --- | --- |
| Development cohort (n = 355) |  |  |
| 39 years (n=3) | 75.0% (19.4–99.4%)  (3/4) | 100% (29.2–100%)  (3/3) |
| 40–49 years (n=8) | 100% (63.1–100%)  (8/8) | 100% (63.1–100%)  (8/8) |
| 50–59 years (n=23) | 95.8% (78.9–99.9%)  (23/24) | 100% (85.2–100%)  (23/23) |
| 60–69 years (n=95) | 86.8% (78.8–92.6%)  (92/106) | 96.8% (91.0–99.3%)  (92/95) |
| 70–79 years (n=142) | 89.0% (83.0–93.5%)  (138/155) | 97.2% (92.9–99.2%)  (138/142) |
| 80 years (n=84) | 94.4% (87.4–98.2%)  (84/89) | 100% (95.7–100%)  (84/84) |

PPV, positive predictive value; SEN, sensitivity; CI, confidence interval.

**Online Resource Table 6** Differences in the diagnostic accuracy of the algorithm for progression phase determination across age brackets in the development cohort.

| Age brackets | Diagnostic accuracy [% (95 % CI)] |
| --- | --- |
| Development cohort (n=348) |  |
| 39 years (n=3) | 100% (29.2–100%)  (3/3) |
| 40–49 years (n=8) | 100% (63.1–100%)  (8/8) |
| 50–59 years (n=23) | 95.7% (78.1–99.9%)  (22/23) |
| 60–69 years (n=92) | 97.8% (92.4–99.7%)  (90/92) |
| 70–79 years (n=138) | 94.2% (88.9–97.5%)  (130/138) |
| 80 years (n=84) | 90.5% (82.1–95.8%)  (76/84) |

CI, confidence interval.

**Online Resource Table 7** Differences in performance metrics of the algorithm in identifying incident gastric cancer cases when the washout period varied from 1 year to 1.5, 2, 2.5, and 3 years.

| washout period | PPV [% (95 % CI)] | SEN [% (95 % CI)] |
| --- | --- | --- |
| 1 year | 89.7% (84.5–93.6%)  (174/194) | 98.3% (95.1–99.6%)  (174/177) |
| 1.5 years | 90.1% (85.0–93.9%)  (173/192) | 97.7% (94.3–99.4%)  (173/177) |
| 2 years | 90.1% (85.0–93.9%)  (173/192) | 97.7% (94.3–99.4%)  (173/177) |
| 2.5 years | 90.1% (85.0–93.9%)  (173/192) | 97.7% (94.3–99.4%)  (173/177) |
| 3 years | 90.1% (85.0–93.9%)  (173/192) | 97.7% (94.3–99.4%)  (173/177) |

PPV, positive predictive value; SEN, sensitivity; CI, confidence interval.
